# Dual-Polarized Dipole Antenna with Wideband Stable Radiation Patterns Using Artificial Magnetic Conductor Reflector

**DOI:** 10.3390/s24123911

**Published:** 2024-06-17

**Authors:** Xianjing Lin, Jielin Mai, Hongjun He, Yao Zhang

**Affiliations:** 1 School of Electronic Engineering and Intelligence, Dongguan University of Technology, Dongguan 523808, China; linxj@dgut.edu.cn (X.L.); 13413325001@163.com (J.M.); hongjun_he77@163.com (H.H.); 2 Institute of Electromagnetics and Acoustics, Xiamen University, Xiamen 361005, China

**Keywords:** wideband antenna, dual-polarized antenna, dipole antenna, radiation pattern restoration

## Abstract

This paper presents a wideband dual-polarized dipole antenna structure operating at 1.7–3.8 GHz (76.4%). For a traditional 4G dipole antenna that covers the band 1.71–2.69 GHz, it is difficult to maintain the satisfactory impedance matching and normal stable radiation patterns within the 5G sub-6 GHz band 3.3–3.8 GHz, mainly due to the fixed antenna height no longer being a quarter-wavelength. To solve this, a connected-ring-shaped metasurface structure is proposed and deployed to operate as an artificial magnetic conductor (AMC). As a result, stable antenna radiation patterns are obtained within the whole band 1.7–3.8 GHz. For verification, this wideband dipole antenna using AMC is implemented and tested. The measured results show that the proposed antenna has an impedance bandwidth of 80.7% (1.7–4.0 GHz). It has an average measured in-band realized gain of 7.0±1.0 dBi and a stable 70±5 half power beam width (HPBW) within the 4G/5G-sub 6GHz bands 1.71–2.69 GHz and 3.3–3.8 GHz.

## 1. Introduction

The dual-polarized dipole antenna has been widely used in the base-station antenna array scenario as the basic antenna array element [1,2,3,4,5]. The antennas, which operate at 1.71–2.69 GHz [1,6,7,8,9,10] and 3.3–3.8 GHz [11] bands, are widely used in sub-6 GHz base-station applications. The antenna designers are devoted to developing wideband antennas that simultaneously cover the above two frequency bands.

However, it is challenging to maintain stable antenna radiation patterns with a fixed antenna height in such a wide frequency band of 1.71–3.8 GHz. Two mainstream approaches are used to solve this problem. One is to design dual-band or notched-band antennas that operate at the two target frequency bands 1.71–2.69 GHz and 3.3–3.8 GHz [12,13]. The other method is to use parasitic components to increase the impedance bandwidth [14,15,16,17,18]. In [14], an extra pair of shorted dipoles were added, and the impedance bandwidth was increased to 74.5% (1.69–3.7 GHz). In [15], parasitic elements such as metal cylinders and triangle metal palates were involved, and the antenna realized a 78.6% bandwidth (1.59–3.65 GHz). However, for the above two designs, the antenna gains at upper-band frequencies (>3 GHz) decreased/increased a lot, indicating unstable radiation patterns. To improve the radiation performance in upper-band frequencies, the design in [16] printed a circular patch and four pairs of dipole strips on an extra substrate layer. Stable radiation patterns over the entire frequency band were realized. However, since this layer substrate is installed above the antenna structure, it inevitably increases the overall antenna profile, which is undesirable for base station applications. Other components such as parasitic posts [17] and partially coupled stubs [18] have also been applied to stabilize the antenna radiation patterns recently. AMC reflectors have been extensively studied for low-profile antenna designs [19,20,21], but they have not been used for the wibeband stable radiation performance. With regard to this problem, this work utilized a connected-ring-shaped metasurface structure as an artificial magnetic conductor (AMC). As a result, a dual-polarized cross-dipole antenna achieved an average in-band realized gain of 7.0 ± 1.0 dBi and a stable 70° ± 5° half power beam width (HPBW) within the band 1.7–3.8 GHz.

## 2. Wideband Antenna Design

### 2.1. Problem of Traditional Wideband Antenna

**Figure 1:**
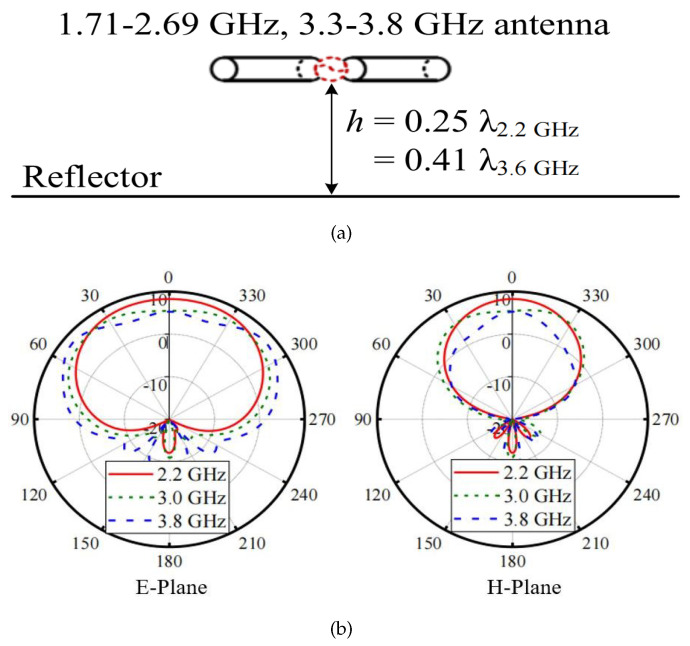
(**a**) Diagram and (**b**) E-/H-plane radiation patterns of a 1.7–3.8 GHz antenna.

Figure 1a displays a traditional directional dipole antenna using a reflector. Suppose this antenna operates at 1.7–3.8 GHz and the antenna height h is set to be a quarter wavelength of 2.2 GHz ($\lambda_{2.2}$ GHz), which is the center operating frequency of the band 1.71–2.69 GHz. As is known, the antenna radiation patterns at upper-band frequencies are distorted since the fixed antenna height is no longer quarter-wavelength at these frequencies. Figure 1b illustrates the antenna radiation patterns at 2.2 GHz, 3.0 GHz, and 3.8 GHz. As can be seen, the radiation patterns at 3.0 and 3.8 GHz are not stable or even distorted. This is because the 3 dB beamwidth increases as the frequency enhances from 1.7 GHz to 2.8 GHz. However, it decreases drastically when the frequency is larger than 3.6 GHz.

**Figure 2:**
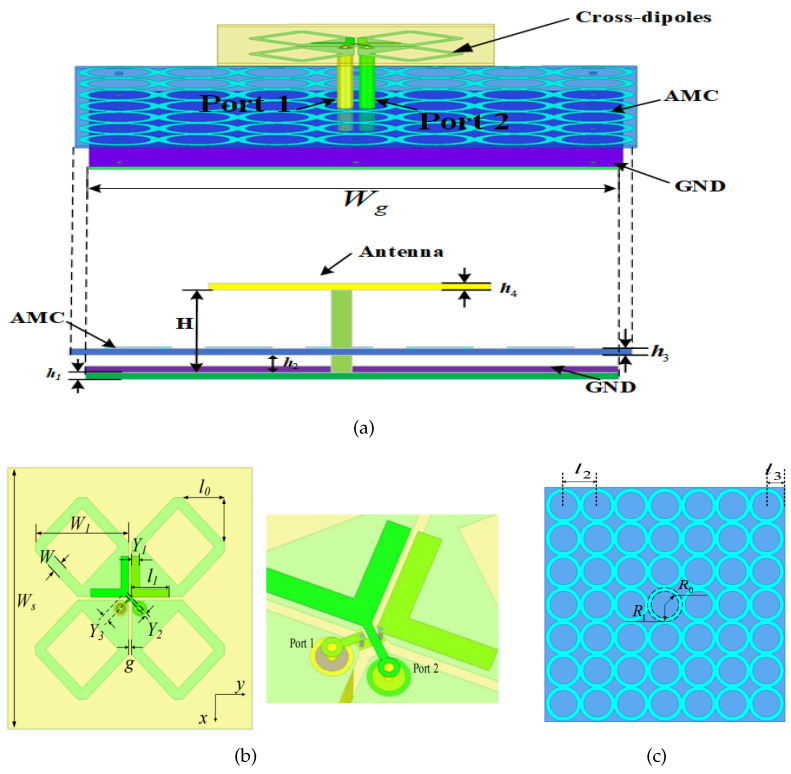
Geometry of the proposed wideband antenna: (**a**) total view, (**b**) dipole antenna, and (**c**) AMC (*H* = 30, $h_{1}$ = 1, $h_{2}$ = 2.5, $h_{3}$ = 0.8, $h_{4}$ = 0.8, $l_{0}$ = 12, $l_{1}$ = 10.9, $l_{2}$ = 20, $l_{3}$ = 11, *W* = 1.7, $W_{s}$ = 70, $W_{g}$ = 135, $W_{1}$ = 26.5, $Y_{1}$ = 2.3, $Y_{2}$ = 0.8, $Y_{3}$ = 2.0, *g* = 1, $R_{0}$ = 8.1, and $R_{1}$ = 10.6, all in mm).

To solve this problem, this letter proposed a wideband dual-polarized dipole antenna with normal stable radiation patterns within the band 1.7–3.8 GHz, as shown in Figure 2. It consists of three parts: a dipole antenna radiator, a connected-ring-shaped metasurface structure as the AMC, and a ground as the reflector. An air layer exists between the AMC reflector and the ground plane. The AMC structure is inserted between the antenna radiator and the reflector, and therefore it does not increase the overall antenna height. The antenna radiators are excited by a pair of Y-shaped feedlines that are directly connected to two coaxial cables. The AMC structure is realized by a 7 × 7 connected ring array. This AMC is deliberately designed to operate at upper-band frequencies (3.3–3.8 GHz) for phase shift compensation. Detailed parameter values are also included in Figure 2. The design procedure and working principle are investigated and revealed in the following part.

### 2.2. Design Procedure

**Figure 3:**
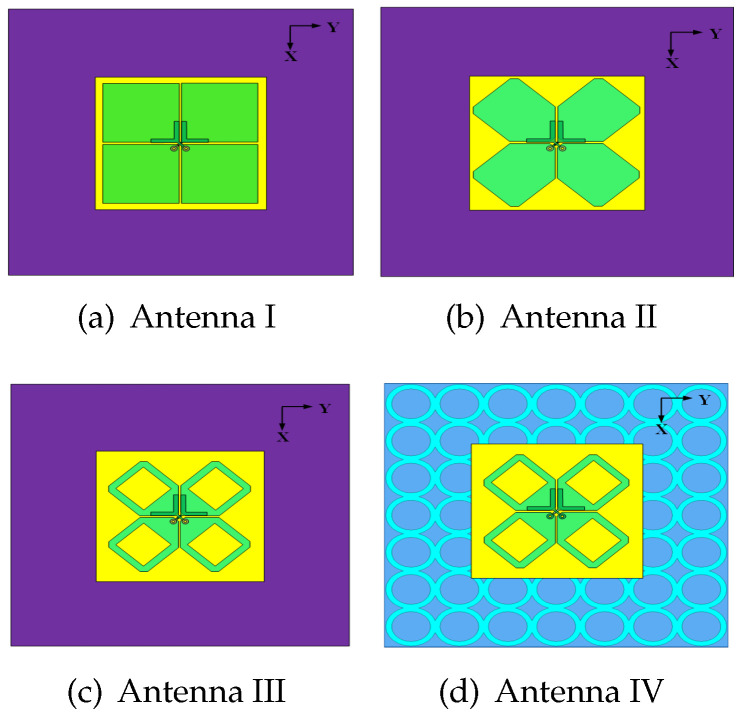
Structures of Antennas (**a**) I, (**b**) II, (**c**) III, and (**d**) IV.

**Figure 4:**
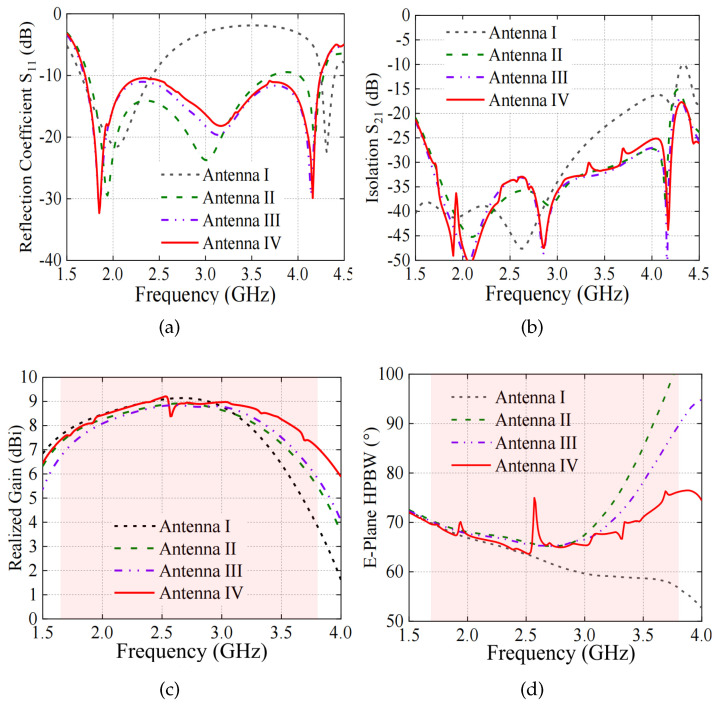
Antenna results including (**a**) reflection coefficient $S_{11}$, (**b**) isolation parameter $S_{12}$, (**c**) realized gain, and (**d**) HPBW.

Figure 3 depicts the structures of four reference antennas, denoted as Antenna I, II, III, IV. The original Antenna I is a dipole antenna with rectangular radiators and Y-shaped coupled feedlines [7]. To enhance the antenna impedance bandwidth and maintain stable radiation patterns, this structure is then modified to Antennas II, III, and IV. The simulation results of the four antennas, including the reflection coefficient $S_{11}$, isolation parameter $S_{12}$, realized gain, and HPBW, are plotted and compared in Figure 4. As observed, Antenna I has limited impedance bandwidth (41.2%, 1.64–2.49 GHz).

However, Antennas II, III, and IV are well matched within the band 1.7–3.8 GHz, and the port-to-port isolation |$S_{12}$| is better than 25 dB. It is noted that the realized gains and HPBW of the four antennas differ a lot, especially at upper-band frequencies (3.0–3.8 GHz). Within the band 1.7–3.8 GHz, the Antenna IV has a stable realized gain ranging from 7.0 dBi to 9.1 dBi. The HPBW is also stable within the range 70° ± 5°.

### 2.3. AMC Working Principle

**Figure 5:**
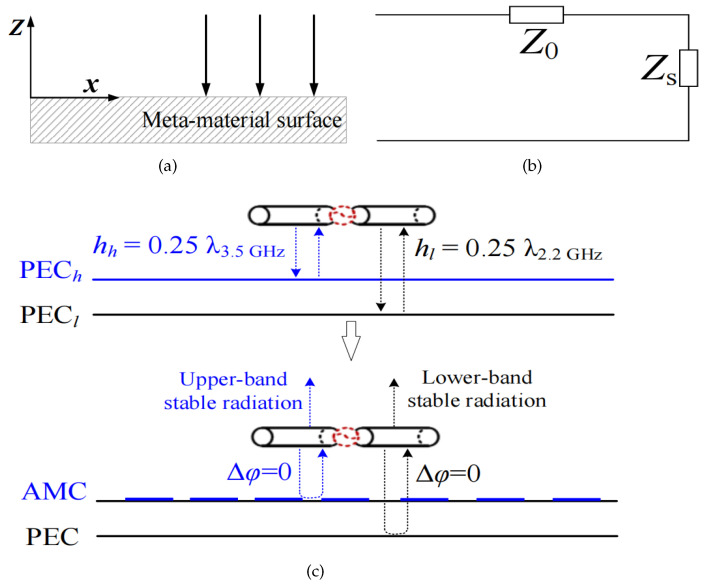
Diagram of Antenna IV’s working principle: (**a**) electromagnetic waves incident on the surface of the material, (**b**) equivalent single port model, and (**c**) constructive interference of the antenna.

For an antenna, the reflector is an important factor affecting its radiation performance, and analyzing the formation principle of its reflection phase characteristics is the key to studying the reflector. Assuming there is an electromagnetic wave that is vertically incident on the reflector, a coordinate system is established based on the material surface, as shown in Figure 5a. The corresponding equivalent model is shown in Figure 5b. The transmission line network can be used to calculate the reflection coefficient:

(1) $$\Gamma =\frac{Z_{S}-Z_{0}}{Z_{S}+Z_{0}}=\left|\Gamma \right|e^{\pm i\phi }$$

(2) $$\phi =im\left\{In\left(\frac{Z_{S}-Z_{0}}{Z_{S}+Z_{0}}\right)\right\}=im\left\{In\left(\frac{Z_{S}-\eta }{Z_{S}+\eta }\right)\right\}$$

where $Z_{S}$ is regarded as the impedance of the material surface, $Z_{0}$ is the impedance of the dipole antenna, and φ is the phase difference between the incident and the reflected wave of the electromagnetic wave. When $Z_{S}=0$, then Γ=1, ϕ=π. It can be seen that when the surface impedance is an ideal electrical conductor, the reflection phase of the reflector is 180°. Only when the distance between the radiator and the reflector is λ/4 can the phase difference with a spatial phase delay of 180° be offset with the 180° phase difference generated by the reflector, so that the reflected wave is in the same direction as the main radiation beam. Thus, enhanced radiation is obtained.

According to the above analysis, when the antenna height is set around λ/4 (λ denotes the wavelength of center frequency 2.2 GHz), the impedance bandwidth for normal stable radiation patterns of a traditional dipole antenna is about 45.4% (1.7–2.7 GHz). To maintain normal stable radiation patterns at 2.7–3.8 GHz, another perfect electric conductor ($PEC_{h}$) is required. This $PEC_{h}$ can be realized by using an AMC, as diagramed in Figure 5c. The surface impedance of an AMC is very high over a certain frequency region, so it is also known as a high-impedance surface, which is a type of frequency-selective surface or meta-material surface.

**Figure 6:**
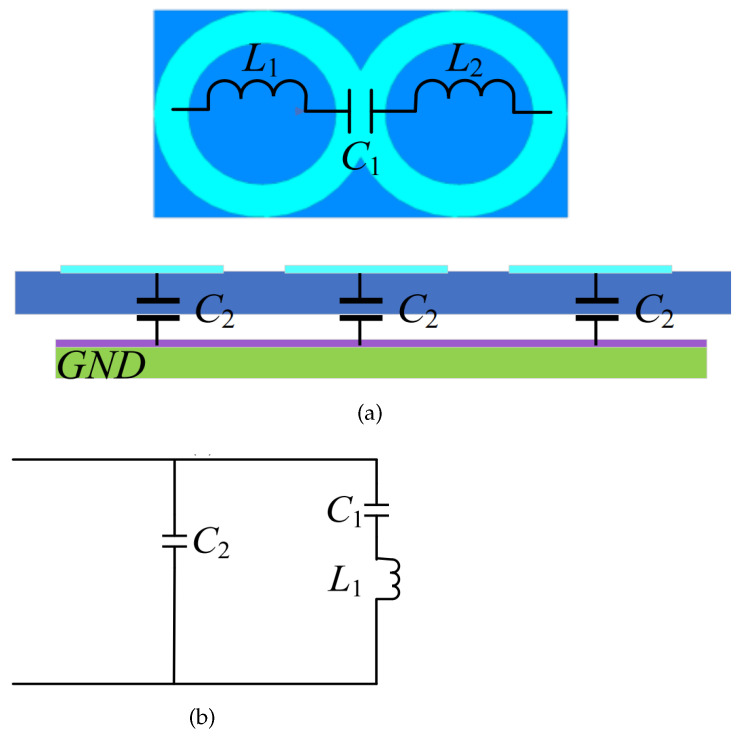
(**a**) Schematic diagram and (**b**) equivalent circuit of the AMC.

The schematic diagram and equivalent circuit of the AMC is shown in Figure 6. The AMC reflector consists of a group of AMC units (metal ring patch) arranged periodically. The capacitor is generated between the metal ring patches, and the inductance is generated between the ground plane and the patches, so the AMC structure can be denoted as a series of LC circuits in parallel. According to the circuit principle, the resonant frequency of the AMC can be obtained as:

(3) $$f=\frac{1}{2\pi \sqrt{\frac{C_{1}C_{2}}{C_{1}+C_{2}}L_{1}}}$$

**Figure 7:**
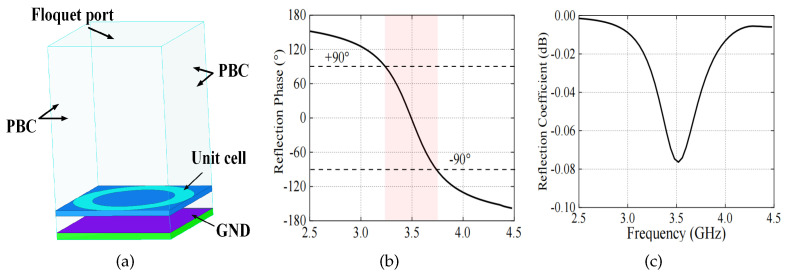
(**a**) Simulation model and (**b**) results including the reflection phase and (**c**) coefficient for determining the reflection properties of the AMC.

To search for the frequency band in which the periodic structure behaves as an AMC, a finite element method (FEM) model is established based on the Bloch–Floquet theory [22]. A single unit cell of the structure, with periodic boundary conditions (PBC) along its four sides, is simulated as seen in Figure 7a to model an infinite periodic surface. It is worth mentioning that the circular ring is chosen as the AMC unit as an example, and other structures, such as square rings, have the same effect. The reflected phase from the periodic surface is normalized to the one from the PEC by

(4) $$\theta =\theta_{FSS}-\theta_{PEC}+\pi$$

The characteristics of the AMC behavior can be verified by calculating the reflection coefficient for a uniform incident plane wave. The phase of the reflection coefficient of an AMC should exhibit a difference of 180° compared to that of a PEC plane. The reflection phase and magnitude of a normally incident plane are shown in Figure 7b. As observed, the reflection phase on the AMC plane varies continuously from −180° to 180° against frequency and is zero at the resonance frequency. The reflection coefficient is larger than −0.1 dB, indicating that almost all the incident waves are reflected. The useful bandwidth of AMC performance is, in general, defined as +90° to −90° on either side of the resonance frequency. The AMC bandwidth can be obtained as

(5) $$BW_{AMC}=\left[\left(f_{up}-f_{lo}/f_{c}\right)\right]\times 100\%$$

where $f_{up}$ and $f_{lo}$ are the frequencies at which the reflection phase equals −90° and 90°, respectively. $f_{c}$ is the center frequency where the reflection phase equals 0°. The AMC frequency bandwidth of the proposed structure is about 16.6% (3.2–3.78 GHz).

**Figure 8:**
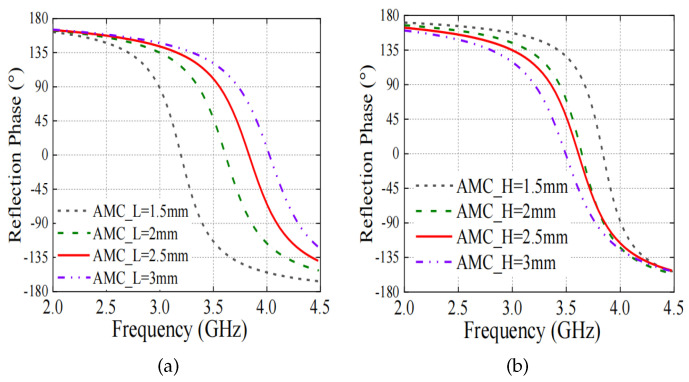
Reflection phase related to different (**a**) unit cell size $AMC_{L}$ and (**b**) unit cell height $AMC_{H}$.

In addition, the operating frequency of the AMC can be shifted to desired values. Figure 8 illustrates the reflection phase of the AMC against the unit cell size $AMC_{L}$, the unit cell height $AMC_{H}$, and the ring dimensions. As seen, the center operating frequency as well as the bandwidth can be easily controlled and optimized. Based on such a structure, the radiation patterns of a dipole antenna within the upper frequency band 2.7–3.8 GHz can be restored without increasing the overall antenna height.

## 3. Dual-Polarized Antenna Implementation

**Figure 9:**
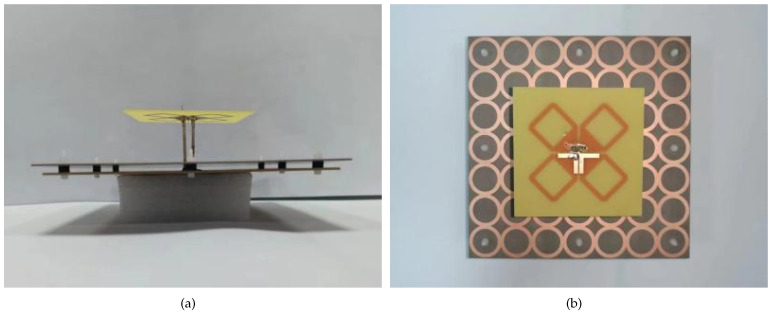
Antenna fabrication prototype. (**a**) side view and (**b**) top view.

**Figure 10:**
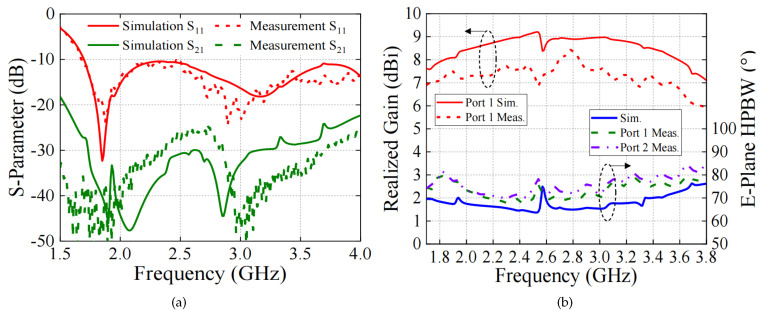
Simulation and measurement results including (**a**) S-parameters and (**b**) realized gains, HPBW.

The proposed dual-polarized antenna was designed, fabricated, and measured, as shown in Figure 9. The optimization was performed using the high-frequency structural simulator (HFSS), and the measurement was accomplished via the Agilent N5227A network analyzer and Satimo system. Figure 10 shows the results including the S-parameters and radiation response. The simulation results agree well with the measurement ones. As seen, the measured bandwidth (|$s_{11}$| < −10 dB) is 76.4% (1.7–3.8 GHz). The measured isolation between two ports within this band is lower than −25 dB, and the average measured in-band gain is about 7.5 dBi. It is 1.5 dBi lower than the simulated gain because of the measured error. A stable 70° ± 5° half power beam width (HPBW) within the band 1.7–3.8 GHz is obtained. There are some abrupt changes in the gain and HPBW curves because of the diffraction effect of the electromagnetic waves at certain frequencies. However, this phenomenon has little impact on the antenna performance.

**Figure 11:**
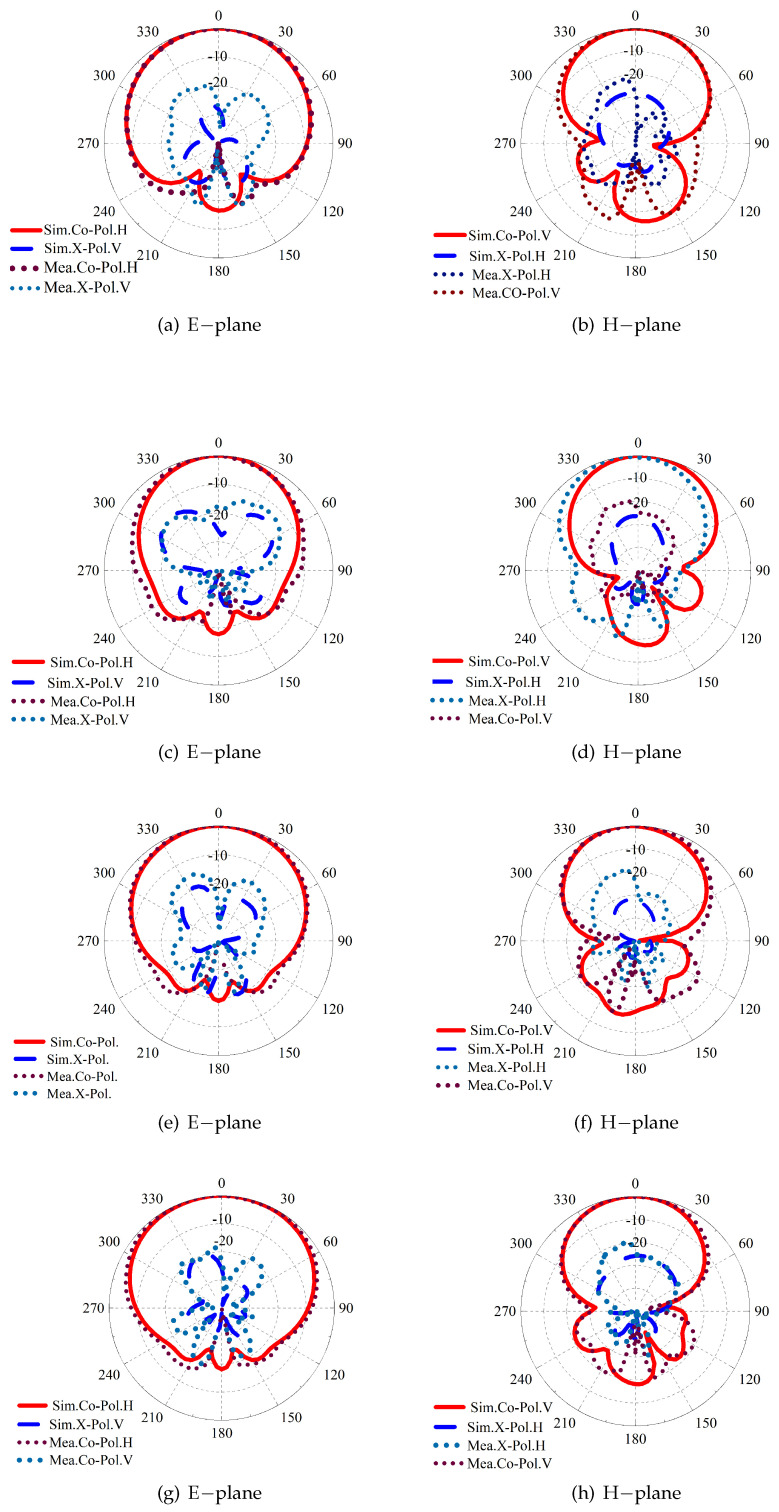
Simulated and measured normalized radiation patterns at (**a**,**b**) 1.7 GHz, (**c**,**d**) 2.7 GHz, (**e**,**f**) 3.3 GHz, and (**g**,**h**) 3.6 GHz.

The antenna radiation patterns at 1.7 GHz, 2.7 GHz, 3.3 GHz, and 3.8 GHz are plotted in Figure 11 when Port1 is excited. As expected, normal stable radiation patterns with low cross-polarization levels are observed. To address the advantages of the proposed work, the comparison results with other related designs are tabulated in Table 1. The dual-band or notched-band antenna concept was adopted in [12,13], and stable radiation patterns were obtained within the target bands 1.71–2.69 GHz and 3.3–3.8 GHz. In [16], an extra superstrate was deployed to print parasitic elements, and thus the antenna overall height was increased. Also, parasitic strips and posts were applied in [18] to achieve a stable realized gain curve. The AMC reflector is used in [19,20,21] to reduce the profile height of the dual-polarized antenna but is not used to achieve wideband (1.7–3.8 GHz) stable radiation performance. Different from the abovementioned methods, by inserting an AMC structure between the antenna radiator and ground, wideband operation and normal stable radiation patterns were realized in the proposed work.

## 4. Conclusions

In this paper, a wideband dual-polarized dipole antenna operating at 1.7–3.8 GHz has been proposed. To restore the distorted antenna radiation patterns within 3.3–3.8 GHz, an AMC has been presented by developing a connected-ring-shaped metasurface or frequency-selective surface structure. This AMC structure was inserted between the antenna radiator and ground, and therefore it did not increase the overall antenna height. The measured results revealed that the proposed antenna simultaneously obtained the wide impedance bandwidth of 76.4%, 7.0 ± 1.0 stable realized gain, normal radiation patterns with 70° ± 5° HPBW, and a simple structure. These merits make the proposed antenna a good candidate for the base station antenna applications.

## Figures and Tables

**Table 1:** Comparison of the wideband dual-polarized antennas.

	Size ($\lambda_{0}^{3}$)	FBW% (BW/GHz)	Implementation	HBPW (°)	Realized Gain (dBi)
[12]	0.8 × 0.8 × 0.26	(47.8%) 1.67–2.72 (16.7%) 3.3–3.9	Parasitic director + baffle	67.5 ± 8.5 64 ± 5	8.1 ± 0.5 8.35 ± 0.25
[13]	0.8 × 0.8 × 0.24	(44%) 1.71–2.69 (7.2%) 3.35–3.6	Notch band antenna	69.5 ± 4 90 ± 10	8.1 ± 0.4 6.6 ± 0.5
[16]	0.8 × 0.8 × 0.22	(100%) 1.7–5.1	Parasitic elements on superstrate	65 ± 5	8.2 ± 0.7
[18]	0.8 × 0.8 × 0.2	(77.7%) 1.7–3.86	Parasitic strips + baffle	72 ± 5	8 ± 0.6
[19]	1.1 × 1.1 × 0.17	(45.4%) 1.7–2.7	AMC	N.A.	4 ± 0.5
[20]	0.9 × 0.9 × 0.13	(19.8%) 3.14–3.83 (23.2%) 4.4–5.02	AMC	N.A.	6 ± 0.8 7 ± 0.5
[21]	3.2 × 3.2 × 0.14	(58.8%) 2.16–3.99	AMC	N.A.	8.8 ± 0.7
**This work**	**0.8 × 0.8 × 0.17**	**(76%) 1.7–3.8**	**AMC**	**70 ± 5**	**7.0 ± 1.0**

$\lambda_{0}$ is the free-space wavelength at the the lowest frequency of the operating bands.

## Data Availability

Data are contained within the article.

## Abbreviations

The following abbreviations are used in this manuscript:

AMC	Artificial magnetic conductor
HPBW	Half-Power Beamwidth
CD	Crossed Dipole
BSA	Base station antenna
